# Intranasal lidocaine for acute migraine: A meta-analysis of randomized controlled trials

**DOI:** 10.1371/journal.pone.0224285

**Published:** 2019-10-23

**Authors:** Pei-Wen Chi, Kun-Yi Hsieh, Kuan-Yu Chen, Chin-Wang Hsu, Chyi-Huey Bai, Chiehfeng Chen, Yuan-Pin Hsu

**Affiliations:** 1 Emergency Department, Wan Fang Hospital, Taipei Medical University, Taipei, Taiwan; 2 Department of Emergency, School of Medicine, College of Medicine, Taipei Medical University, Taipei, Taiwan; 3 Department of Medical Education, National Taiwan University Hospital, Taipei, Taiwan; 4 Department of Public Health, School of Medicine, College of Medicine, Taipei Medical University, Taipei, Taiwan; 5 Cochrane Taiwan, Taipei Medical University, Taipei, Taiwan; 6 Division of Plastic Surgery, Department of Surgery, Wan Fang Hospital, Taipei Medical University, Taipei, Taiwan; 7 Evidence-based Medicine Center, Wan Fang Hospital, Taipei Medical University, Taipei, Taiwan; 8 Research Center of Big Data and Meta-Analysis, Wan Fang Hospital, Taipei Medical University, Taipei, Taiwan; University of Mississippi Medical Center, UNITED STATES

## Abstract

**Background:**

Intranasal lidocaine has been shown to be effective in treating patients with acute migraines; however, its efficacy is still controversial. In this study, we intend to assess the efficacy and safety of intranasal lidocaine compared with a placebo or an active comparator for the treatment of migraines.

**Method:**

PubMed, EMBASE, Cochrane library, and Scopus databases were searched from their inceptions to November 2018. Randomized controlled studies investigating the efficacy of intranasal lidocaine compared with a placebo or an active comparator were selected. Two reviewers independently extracted and synthesized data using a random-effects model. The primary outcome was pain intensity. The secondary outcomes were success rate, the need for rescue medicine, and relapse occurrences. We registered the study at PROSPERO with an ID of CRD42018116226.

**Results:**

Six studies (n = 613) were eligible for the meta-analysis. Overall, the results revealed that the study population who was administered intranasal lidocaine had a lower pain intensity at 5 min (standardized mean difference (SMD) = -0.61; 95% CI = -1.04 to -0.19) and 15 min (SMD = -0.72; 95% CI = -1.14 to -0.19), had a higher success rate (RR = 3.55; 95% CI: 1.89 to 6.64) and a less frequent need for rescue medicine (RR = 0.51; 95% CI = 0.36 to 0.72) than the control group. These beneficial effects were not observed when an antiemetic was administered. Furthermore, intranasal lidocaine use had no significant influence on the relapse rate (RR = 0.89; 95% CI = 0.51–1.56), regardless of the use of antiemetics. Using lidocaine caused local irritation in up to 49.4% of the patients in one report but did not cause major adverse events.

**Conclusion:**

Intranasal lidocaine can be considered a useful option for patients with an acute migraine. It yields a high success rate, a low pain intensity, an infrequent need for rescue medicine, and tolerable adverse events. The administration of antiemetics is an important confounding factor.

## Introduction

A migraine is an episodic disorder characterized by a disabling headache generally associated with nausea, with or without light and sound sensitivity. The prevalence of migraines in the United States ranges from 6% to 9% for men and 18% to 26% for women.[1, 2] In 2009, approximately 44.5 million U.S. adults experienced an episode of a migraine.[3] More than 1.2 million migraine patients visit the United States emergency departments (ED) annually.[4] Migraines have a negative impact on the quality of life of individuals, reduce in workplace productivity, and limit participation in and the enjoyment of social and leisure activities.[5]

Medications commonly used as an abortive treatment for acute migraine include nonsteroidal anti-inflammatory drugs (NSAID), antiemetics, triptan, and ergotamine. Even though these treatments are available, many patients continue to experience poor symptom control. Moreover, these drugs may have serious side effects such as gastrointestinal bleeding with NSAIDs, tardive dyskinesia with antiemetics, the development of serotonin syndrome with triptans, and vascular occlusion and rebound headaches with ergotamine. Therefore, a need exists for an acute migraine intervention that can deliver rapid, complete, and sustained headache relief without causing side effects.[6]

Intranasal lidocaine, a sodium channel blocker and local anesthetic, is considered effective in treating acute migraines by blocking the sphenopalatine ganglion. The sphenopalatine ganglion is a parasympathetic ganglion that lies behind a layer of mucosa in the posteromedial aspect of the nasal cavity and regulates cranial parasympathetic outflow through the release of neuropeptides. Intranasal lidocaine controls migraine pain by inactivating or desensitizing the intracranial nociceptors that contribute to the vasodilation of the cerebral vasculature, producing migraine.[7–9] In 2015, American Headache Society provided a level C recommendation (i.e., inadequate evidence) to use intranasal lidocaine[10], and the Canadian Headache Society weakly recommended (i.e., based on a low level of evidence) the use of intranasal lidocaine[6]. However, recent randomized controlled trials (RCTs) using intranasal lidocaine have shown controversial findings.[11–13]

Due to the equivocal findings in previous studies, it is necessary to determine whether intranasal lidocaine reduces less pain intensity and increase the rates of short-term and sustained headache relief more than a placebo does among patients who present with an acute migraine. Additionally, the administration of a comedication may have confounded the results from previous studies. In this meta-analysis, we intend to synthesize the data from RCTs, assess the efficacy of intranasal lidocaine for acute migraines, and explore potential confounding factors.

## Materials and methods

We followed the preferred reporting items for systematic reviews and meta-analyses (PRISMA) guidelines for this meta-analysis (S1 Checklist).[14] The systematic review was approved by PROSPERO, the online international prospective register of systematic reviews funded by the National Institute for Health Research (PROSPERO ID: CRD42018116226). Ethical approval and patient consent were not required because the present study is a review of previously published articles. We previously published our study protocol in a peer-review journal.[15]

### Search strategy and eligibility of included studies

We used the following keywords to search the PubMed, EMBASE, Cochrane library, and Scopus databases: lidocaine, xylocaine, intranasal, trans-nasal, headache, and migraine (S1 Table). The “related articles” option in PubMed was used to broaden the search. We applied neither language restrictions nor other limitations. We manually checked the references of the available eligible studies for additional potential studies. We also explored the ClinicalTrials.gov registry (http://clinicaltrials.gov) for any associated ongoing or unpublished studies. The last literature search was performed in November 2018. The study design included all RCTs conducted in humans. We excluded cohort studies, case series, and case reports. The target population was acute migraineurs. There were no restrictions on the migraine type (e.g., migraine with aura, migraine without aura), duration of migraine, or frequency of the attack. Studies that had a target population composed of primary headache subjects, and analyzed a subset of the migraine subjects were included. The intervention was the use of intranasal lidocaine through any applicator. The patients in the control group were treated with a placebo or an active comparator. Our primary outcome of interest was pain intensity, measured using a visual analogue scale (VAS), numerical rating scale (NRS), or verbal rating scale (VRS). The secondary outcomes were the success rate, the need for rescue medicine, relapse, and adverse events associated with the intervention or control condition. Initially, duplicate reports were removed. Two authors (PWC and KCWC) independently screened the titles and abstracts to exclude irrelevant studies based on the inclusion criteria. The two authors then independently verified the eligibility of these potentially relevant articles after reviewing the full texts. We used Cohen’s unweighted kappa statistics to measure the degree of agreement between the two independent reviewers.[16] Disagreements in the study selection process between PWC and KCWC were resolved through discussion or by consulting a third senior independent reviewer (CC).

### Data extraction

Two reviewers (PWC and KCWC) independently extracted the relevant details: (1) publication details, including first author, publication year, country; (2) characteristics of the study population; (3) number of participants; (4) regimens of each comparison; (5) eligible outcome data; and (6) time of follow-up. If controversies about the recorded data existed, we resolved them through discussion between both authors (PWC and KCWC) or by consulting a third reviewer (CC).

### Quality assessment

Two authors (CHB and YPH) independently examined the quality of the included studies with the Revised Cochrane risk of bias tool (RoB 2.0) for RCTs.[17] Six domains were assessed, including bias arising from the randomization process, bias due to deviations from the intended intervention, bias due to missing outcome data, bias in measurements of outcomes, bias in selection of the reported result, and other biases. We classified each domain on the study level as either low risk, some concern of risk, or high risk of bias. If there were any disagreements, a third and senior author was involved in resolving them. We summarized the results in a risk of bias graph.

### Statistical analysis

For dichotomous outcomes, we calculated the risk ratio (RR) with 95% confidence intervals (CIs). We used mean differences (MDs) and 95% CIs to measure continuous outcomes. We used standardized mean difference (SMD) and 95% CIs when the continuous data were given on different scales. If the mean and variance were not reported, we estimated the values from the sample size, median, and range. TheI^2^ statistic was used to evaluate heterogeneity among the studies with predetermined thresholds for low (25%–49%), moderate (50%–74%), and high (>75%) levels.[18] We explored possible clinical heterogeneity with prespecified subgroup analyses according to the use of comedication. Publication bias was not assessed because this study included fewer than ten studies. We performed the meta-analysis by using Review Manager, version 5.3.5 (Rev-Man, The Cochrane Collaboration, Oxford, United Kingdom), and the DerSimonian and Laird random-effects model. In addition, pain intensity over time was computed by using GraphPad Prism version 5 (GraphPad Software, San Diego, USA). If a two-sided *p*-value was smaller than 0.05, the difference between the groups was considered significant.

## Results

### Study selection and study characteristics

**Fig 1:**
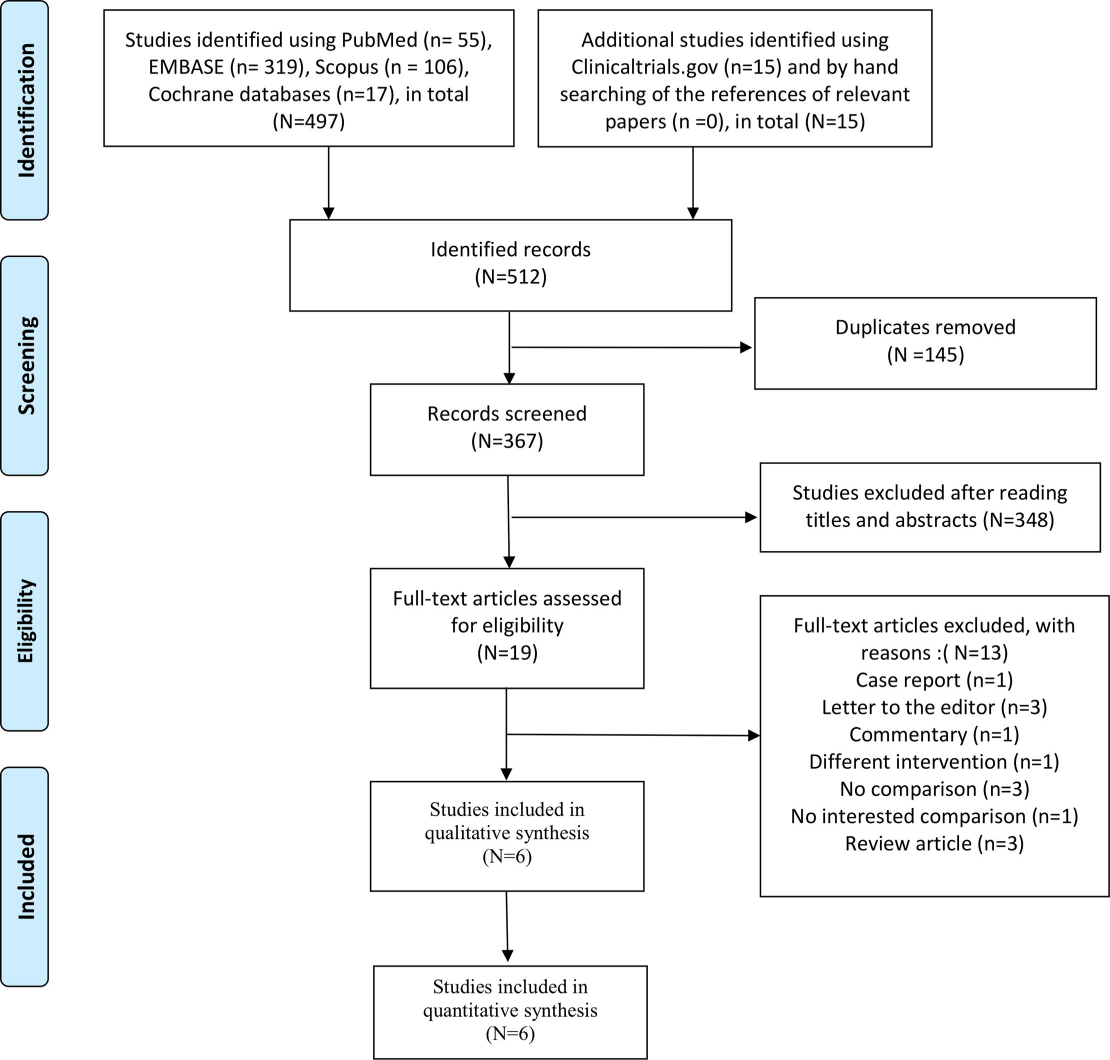
Flow diagram of the search process and search results.

Fig 1 shows the screening and selection process of the studies. Our initial search yielded 512 records. After duplicates were removed (n = 145)and the titles and abstracts were screened (n = 348), 19 full-text articles remained. Of these studies, a case report (n = 1), letters to editor(n = 3), a commentary (n = 1), a study that included different interventions (n = 1), studies that did not include a comparison(n = 3), a study that did not include a relevant comparison(n = 1) and review articles (n = 3) were excluded. Six eligible studies were included in the qualitative and quantitative synthesis.[7, 8, 11–13, 19] Interobserver agreement (κ) for study selection was nearly perfect (κ = 0.85, 95% CI [0.73, 0.98]).

The characteristics of the included studies are summarized in Table 1. All participants with migraines met the International Headache Society (IHS) criteria. Two studies included patients with condition other than migraines; one included patients with primary headaches and another included both patients with primary headaches and those with secondary headaches. Most of the studies excluded participants with pregnancy, lactation, or a sign of a secondary headache (S2 Table). Four studies mentioned that participants who took analgesics 2 or 6 hours before being admitted to the ED were excluded. Sample sizes ranged from 49 to161 patients, with a mean age ranged from 29.6 to 47.1 years. All of the studies enrolled more females than males. The volume or concentration of the lidocaine solution administered to the patients varied. Four studies mentioned the administration of the drug by the Barre method via drops, a spray, or pump devices. There were variations in the administration of comedications. Three of the included studies used intravenous antiemetic agents, including prochlorperazine[8], metoclopramide[11], or chlorpromazine[12]. The duration of the follow-up period ranged from 30 minutes to 1 month.

**Table 1:** Characteristics of the included trials.

Study	Included criteria	Age:	Participants (%females)	Intervention		Follow up
				Regimens and routes	Barre method	
Maizel 1996 [19]	Age > 18 years; migraine with or without aura (IHS criteria); at least moderate intensity	I: 43 (34–50) * C: 40 (31–47) *	I: 53 (87%) C: 28 (75%)	I: 0.5 mL of 4% lidocaine C: saline	Yes	24 hours
Maizel 1999 [7]	Age, 18–65 years; migraine with or without aura (IHS criteria); migraine frequency 1–6 times per month;	I: 44.5 (9.1) C: 47.1 (10.2)	I: 66 (83%) C: 65 (88%)	I: 0.5 mL of 4% lidocaine C: saline	Yes	1 month
Mohammadkarimi 2014 [13]	Age ≧14 years; primary headache (IHS criteria; migraine, tension, and cluster); secondary headaches	I: 33.5 (13.3) C: 37.2 (14.6)	Participant in total: 90 (58%)	I: one puff of 10% lidocaine into each nostril C: saline	NR	30 mins
Blanda 2001 [8]	Age, 18–50 years; migraine with or without aura (IHS criteria)	NR	I: 27 (85%) C: 22 (86%)	I: 2 mL 4% lidocaine + IV 10 mg prochlorperazine C: saline + IV 10 mg prochlorperazine	Yes	24 hours
Avcu 2017 [11]	Age >18 years; migraine (IHS criteria)	I: 36.0 (12.0) C: 35.0 (11.0)	I: 81 (69%) C: 81 (85%)	I: 10% lidocaine + IV 10 mg metoclopramide C: saline + IV 10 mg metoclopramide	Yes	24–72 hours
Barzegari 2017 [12]	Age, 15–55 years; primary headache (met IHS criteria; migraine: 32%, tension headache: 22%, cluster headache: 46%)	I: 33 (8.5) C: 29.6 (8.6)	I: 50 (56%) C: 50 (52%)	I: 1 ml intranasal lidocaine 2% + IV 7.5 mg chlorpromazine C: saline + IV 7.5 mg chlorpromazine	NR	1 hour

C, control; ED, emergency department; I, intervention; IHS, international headache society; IV, intravenous; NR, not reported

*, median (IQR).

### Risk of bias in the included studies

**Fig 2:**
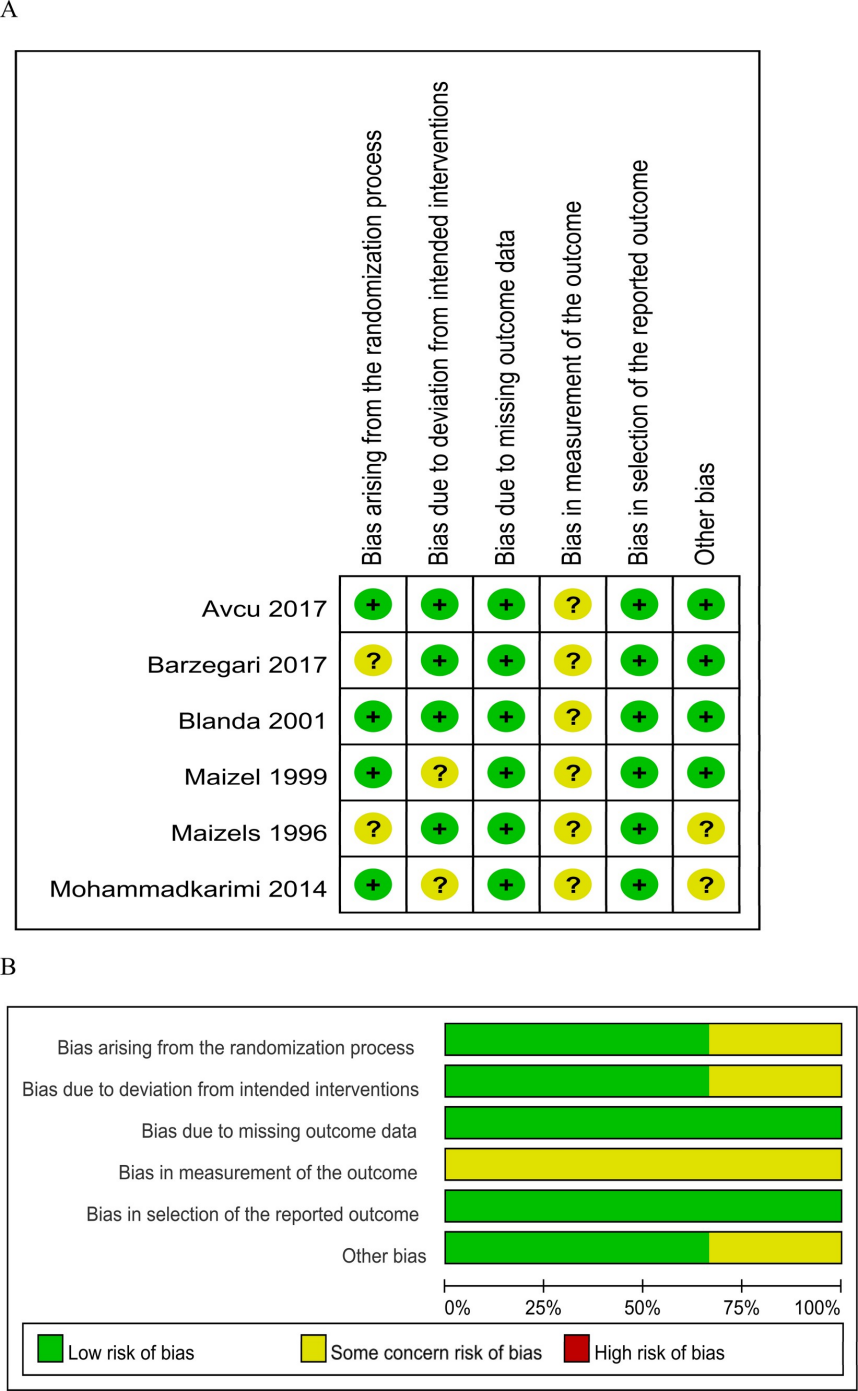
Methodological quality: (A) risk of bias summary of the randomized controlled trials; (B) risk of bias graph of the randomized controlled trials.

The results of the risk of bias assessments are displayed in Fig 2. All studies had a low risk of bias for missing outcome data and selective outcome reporting but had some concern risk of bias for the measure of the outcome because the pain score is a patient-reported outcome. Two studies were rated as having some concern of a risk of bias arising from the randomization process because no information about allocation and concealment was provided. We rate two studies as having some concern risk of bias for the deviation from the intended intervention because there was no blinding process or per-protocol analysis. Two studies were rated as having some concern of a risk of other bias because no calculations of a prespecified sample size.

### Primary outcomes

#### Pain intensity

**Fig 3:**
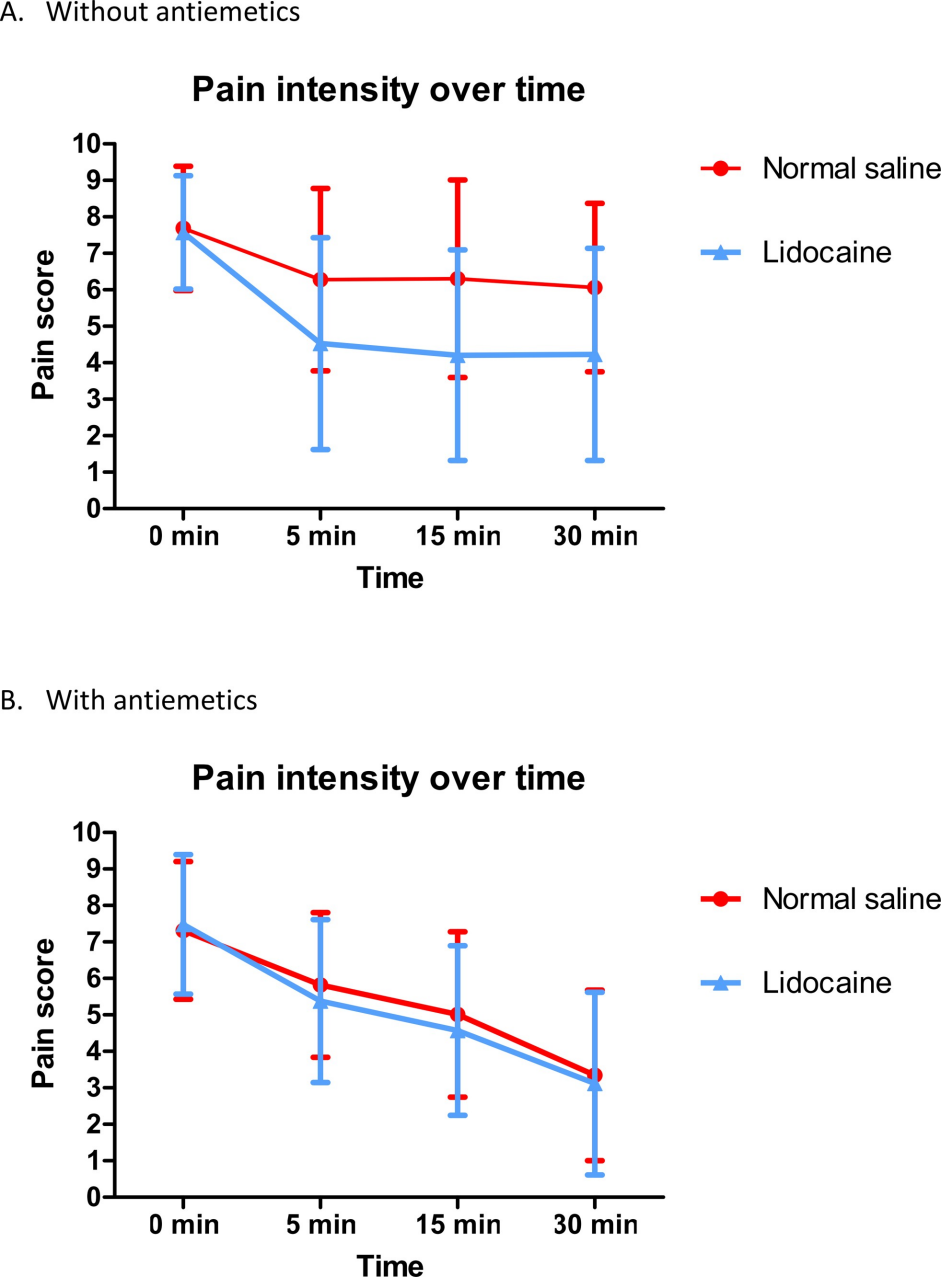
**The pain intensity decreased over time: (A) without antiemetics; (B) with antiemetics.** Error bars represent the standard deviation of each study, and the average from each study is represented by a single data point.

A total of 5 studies assessed pain intensity using a different scale. Two studies used the NRS[7, 11]; 3 studies used the VAS[8, 12, 13]; one used a 0–5 pain scale[19]. There was no significant difference in the baseline pain intensity between the groups. The pain intensity at 5 min and 15 min after treatment favored intranasal lidocaine compared with the control (Table 2, SMD_5 min_ = -0.45, 95% CI [-0.71, -0.19], p < 0.05; SMD_15 min_ = -0.41, 95% CI [-0.72, -0.09], p < 0.05). The results of the pooled studies were homogeneous at 5 mins and heterogeneous at 15 mins (I^2^_5 min_ = 2%; I^2^_15 min_ = 54%). A subgroup analysis indicated that a decrease in pain intensity at 5 mins and 15 mins was observed only in the comedication without antiemetics group (Table 2, SMD_5 min_ = -0.61, 95% CI [-1.04, -0.19]; SMD_15 min_ = -0.72, 95% CI [-1.14, -0.29]) and not in the antiemetic comedication group. The pain intensity over time is displayed in Fig 3. The result showed that using comedication with or without antiemetics was a confounding factor.

**Table 2:** Pooled results of pain intensity.

Time	Subgroups	No of studies	No of patients	SMD [95% CI]	*p-*value	Heterogeneity (I^2)^
0 min	Overall	5	410	0.05 [-0.15, 0.25]	0.61	0
	Without antiemetic	2	99	-0.11 [-0.52, 0.30]	0.60	0
	With antiemetic	3	301	0.10 [-0.13, 0.32]	0.39	0
5 min	Overall	4	248	-0.45 [-0.71, -0.19]	< 0.05^*^	2
	Without antiemetic	2	99	-0.61 [-1.04, -0.19]	< 0.05^*^	0
	With antiemetic	2	149	-0.32 [-0.78, 0.14]	0.17	46
15 min	Overall	5	410	-0.41 [-0.72, -0.09]	< 0.05^*^	54
	Without antiemetic	2	99	-0.72 [-1.14, -0.29]	< 0.05^*^	0
	With antiemetic	3	311	-0.28 [-0.67, 0.10]	0.15	62
30 min	Overall	4	329	-0.19 [-0.61, 0.24]	0.39	66
	Without antiemetic	1	18	-0.47 [-1.43,0.49]	0.34	NA
	With antiemetic	3	311	-0.14 [-0.64, 0.35]	0.58	77

CI, confidence interval; NA, not applicable; SMD, standardized mean difference

*, statistically significant

### Secondary outcomes

#### Success rate

**Fig 4:**
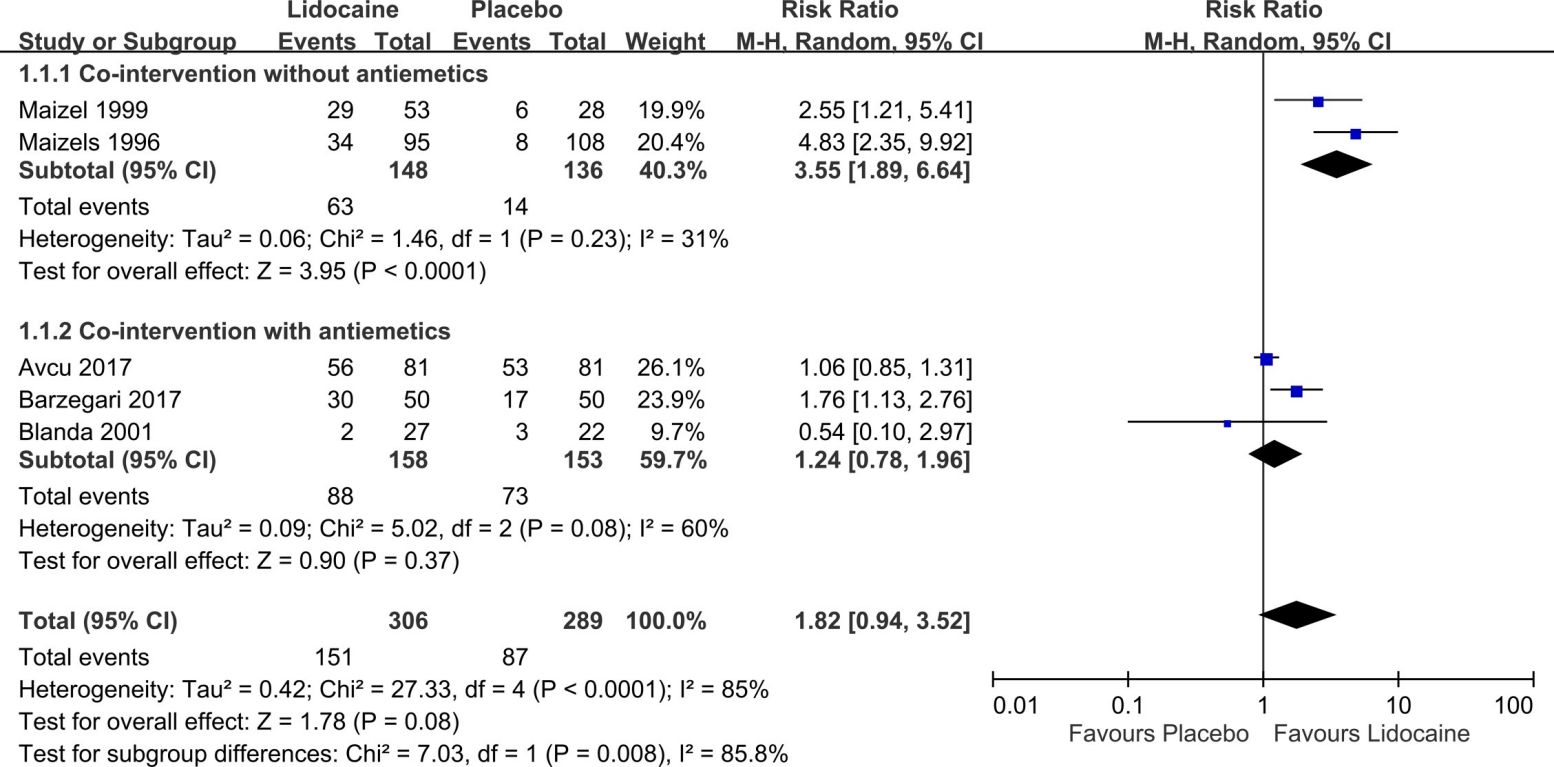
Forest plot of the success rate.

Five studies (n = 595) evaluated the success rate[7, 8, 11, 12, 19]. The definition of success rate varied across these studies; One study defined success rate as the “relief of headache: mild or none”[7]; two defined it as headaches diminished by at least 50%[11, 19]; one defined it as a decrease in the pain score by at least 3 points decrease of pain score[12]; and one defined it as a decrease by 50% or more in the initial pain score or an absolute pain score of 2.5 cm or less[8]. The results showed that intranasal lidocaine yielded a 1.82 times higher success rate than the control did (Fig 4, pooled RR: 1.82; 95% CI: 0.94 to 3.52; I^2^ = 85%). However, the effect was insignificant, and heterogeneity was high. A subgroup analysis indicated that in patients who did not receive antiemetic comedication, intranasal lidocaine produced a significantly higher success rate than the control condition, which was 3.55 times higher than that of the control condition (Fig 4, pooled RR: 3.55; 95% CI: 1.89 to 6.64; I^2^ = 31%). The effect was not observed in the group of patients receiving antiemetic comedication (Fig 4, pooled RR: 1.24; 95% CI: 0.78 to 1.96; I^2^ = 60%)

#### The need for rescue medicine

**Fig 5:**
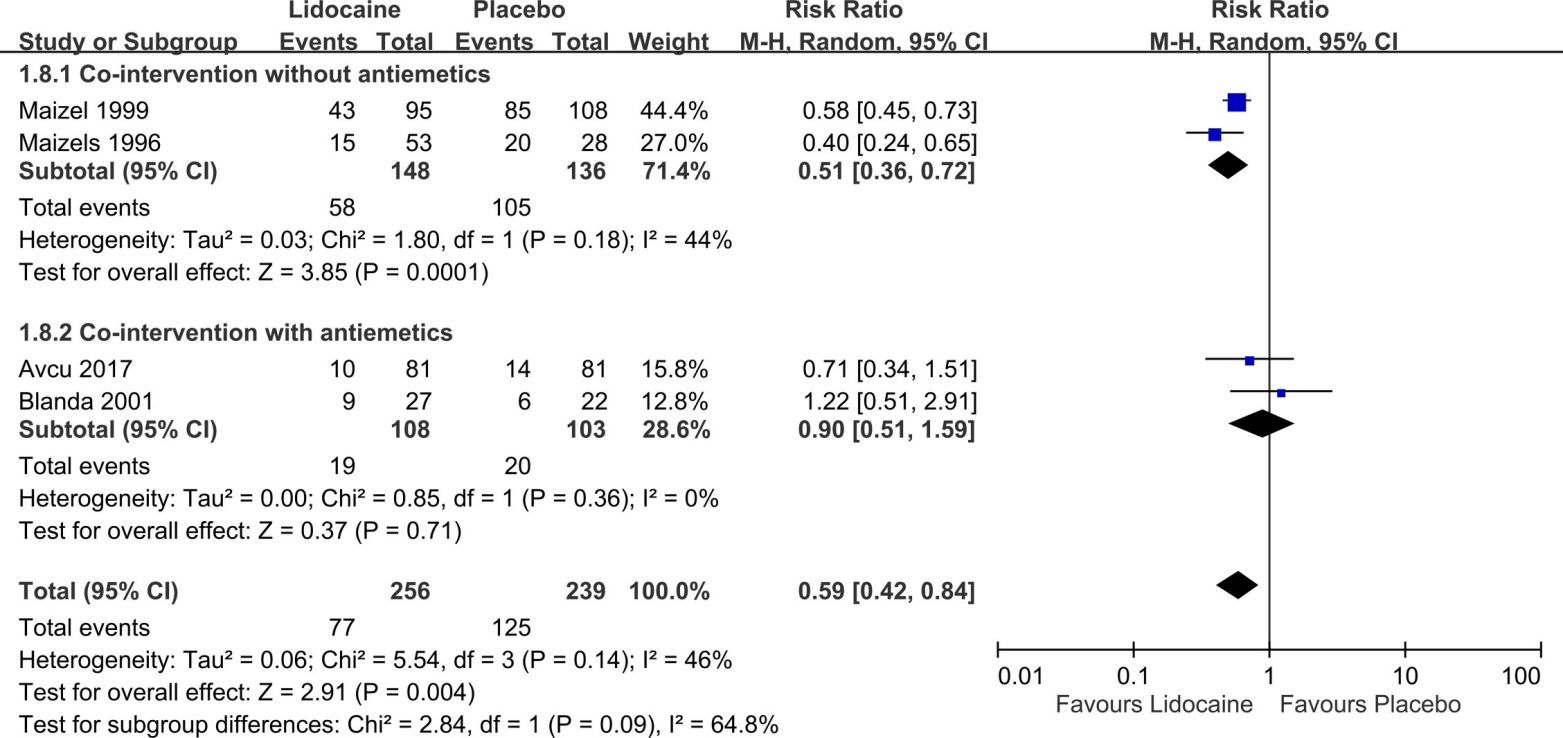
Forest plot of the need for rescue medicine.

Five studies (n = 495) investigated the need for rescue medication. The results indicated that the intranasal lidocaine group had a significantly lower risk for needing rescue medicine than the control group did (Fig 5, pooled RR: 0.59; 95% CI: 0.42 to 0.84; I^2^ = 64.8%), but the heterogeneity was high. A subgroup analysis showed that in patients who did not receive antiemetic comedication, the intranasal lidocaine group maintained a relatively lower risk for needing rescue medicine compared with the control group (Fig 5, pooled RR: 0.51; 95% CI: 0.36 to 0.72; I^2^ = 44%); however, in patients receiving antiemetic comedication, the beneficial effect was not significant (Fig 5, pooled RR: 0.90; 95% CI: 0.51 to 1.59; I^2^ = 0%).

#### Relapse

**Fig 6:**
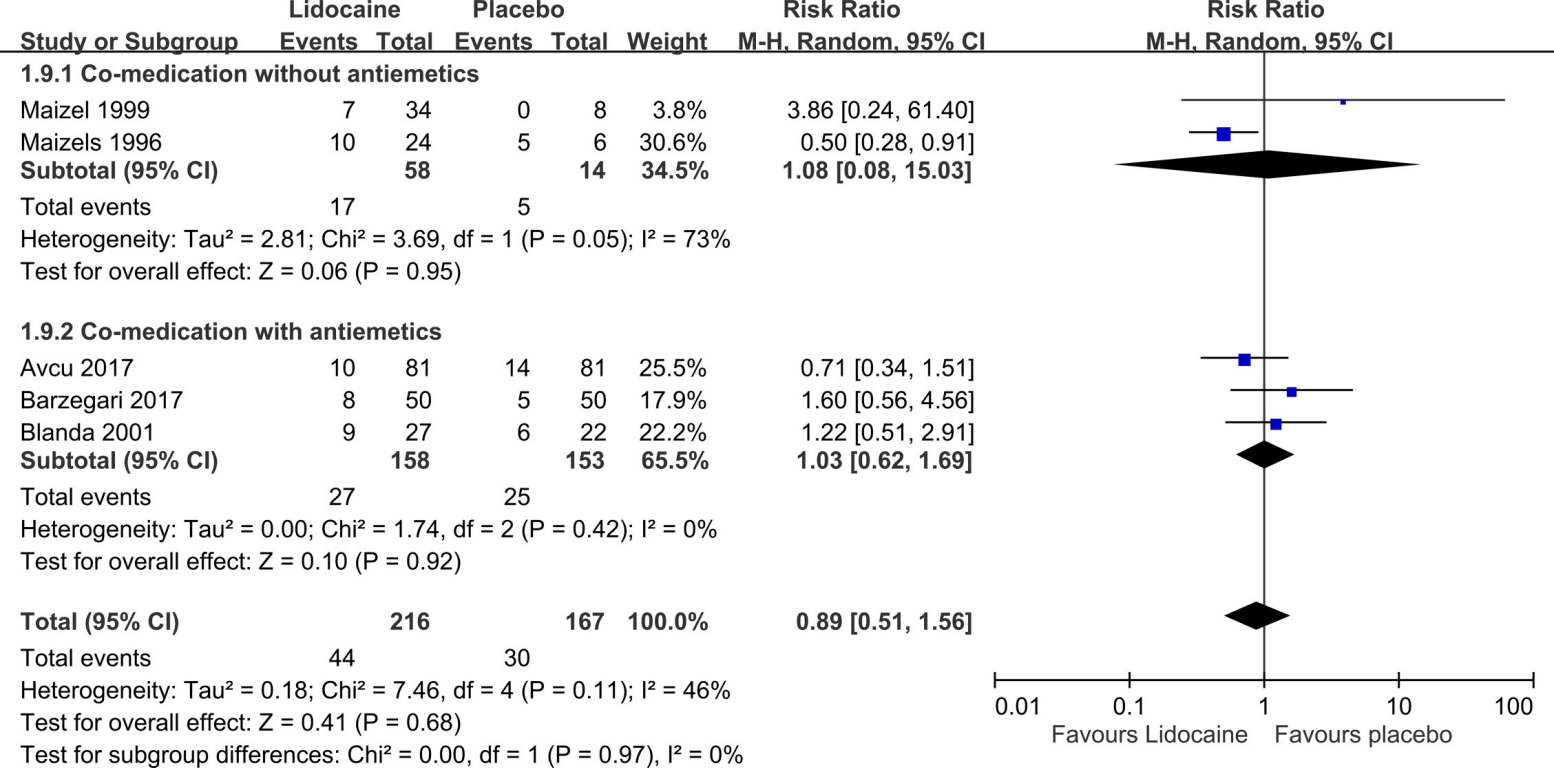
Forest plot of relapse.

Five studies (n = 383) evaluated the relapse of a headache. The pooled RR indicated no significant decrease in relative risk favoring intranasal lidocaine over the control condition (Fig 6, pooled RR: 0.89, 95% CI:0.51 to 1.56, I^2^ = 46%). A subgroup analysis demonstrated that in patients who received intranasal lidocaine with/without antiemetics, the results consistently showed no significant decrease in the relative risk for the relapse of a headache.

#### Safety outcome

The result of the safety outcome is summarized in Table 3. Local symptoms, including burning or numbness in the nose or in and around the eye, an unpleasant taste and irritation of the throat, were noted almost exclusively in the lidocaine group. Avcu et al. reported that the proportion of patients with local symptoms was as high as 49.4%.[11] Additionally, Blanda et al. reported that up to 20.4% of the patients who received antiemetics developed akathisia.[8]

**Table 3:** Summary of findings for safety outcome.

Intervention	Reference	Findings
Lidocaine vs. placebo	Maizels et al. [19]	1. Adverse effects were limited to local symptoms of burning or numbness in the nose or in and around the eye. 2. The use of intranasal lidocaine often caused an unpleasant taste, numbness in the throat and a sensation of gagging.
	Mohammadkarimi et al. [13]	Not mentioned
	Maizels et al. [7]	1. Adverse effects were limited to a local irritation (burning, stinging, numbness) of the nose or eye (n = 101/203); unpleasant taste, gagging, and numbness of the throat (n = 16/203); and nausea (n = 13/203). 2. No severe adverse effect.
Lidocaine + antiemetics vs normal saline + antiemetics	Blanda et al. [8]	1. There was no adverse reaction to the administration of nasal lidocaine. 2. Physicians administered diphenhydramine for akathisia in six of 27 from the lidocaine group and four of 22 from the placebo group cases 3. No dystonic events were recorded.
	Barzegari et al.[12]	Not mentioned
	Avcu et al.[11]	1. 40 patients in lidocaine group (49.4%) reported a transient irritation in their noses, whereas 9 in the saline solution group (11.1%) experienced it. 2. No serious adverse events, including anaphylaxis, akathisia, dystonia, and seizure, were reported in either group.

## Discussion

The results of the present review indicated that the group of patients with acute migraines who received intranasal lidocaine had less pain intense at 5 min and 15 min, a higher success rate, and a lower need for rescue medicine for than did the control group. Whether patients received antiemetics is an important confounding factor. Intranasal lidocaine had no significant influence on the relapse rate compared with the control condition, regardless of the use of antiemetics. The use of intranasal lidocaine caused local nasal irritation.

More than 1 million patients present to the ED in the United States annually to obtain relief from acute migraines.[4] Sustained headache relief remains elusive; numerous medications have been examined to treat severe migraine in the ED.[4, 20] Intranasal lidocaine has been regarded as a critical migraine intervention that can deliver rapid, complete, and sustained headache relief.[19, 21] However, the findings from the literature showed mixed results.[7, 8, 11–13, 19] In our meta-analysis, we found that using intranasal lidocaine produced lower the intensity at 5 min according to the SMD and 15 min according to the SMD compared to using saline. Based on the standard deviation in the control group of a study[7] (SD_5min_ = 2.6; SD _5min_ = 2.8), this result is equivalent to a decrease by −1.6 (at 5 min) and −2.0 (at 15 min) on a scale from 0 to 10, meaning that these effects are clinically significant.[22] Furthermore, we also found that compared with saline, intranasal lidocaine was associated with a higher success rate (42.6% vs. 10.3%) and a lower need for rescue medicine (39.2% vs. 77.2%). In a noncontrolled study, Kudrow et al. found that 12 out of 23 migraine patients had complete headache relief after using 4% intranasal lidocaine; the effect was sustained at 24 hours.[21] In another retrospective study, Binfalah et al. found that using intranasal 2 cc of 2% lidocaine through the Sphenocath device decreased mean NRS scores from 6.8 at baseline to 0.9, 0.6, and 0.8 at 15 minutes, 2 hours, and 24 hours after the procedure, respectively[23]; 70.9%, 78.2%, and 70.4% of migraine patients (n = 55) were completely headache-free at 15 minutes, 2 hours, and 24 hours.[23] In a randomized, double-blind, placebo-controlled study, Schaffer et al. used bupivacaine or a normal saline solution delivered intranasally (0.3 mL per side) with the Tx 360® device for patients with acute anterior frontal headaches without a specific classification.[24] The results indicated that there were no differences between the two groups in the percentage of patients that experienced a 50% reduction in the headache score at 15 minutes. However, more patients in the bupivacaine group than in the saline group were headache free (72.2% vs. 47.5%), and nausea free (94.4% vs. 77.5%) at 24 hours.[24] The reason is that it may take longer than 15 minutes for bupivacaine to to have the desired effect on the sphenopalatine ganglion in some patients. Moreover, Cady et al. conducted a placebo-controlled study in which intranasal 0.5% bupivacaine (n = 26) versus saline (n = 12) was administered through the Tx 360® device twice a week for six weeks for an acute treatment of chronic migraines. The results showed that intranasal bupivacaine led to a reduction in the numeric rating scores for pain at 15 minutes, 30 minutes, and 24 hours after each treatment.[25] The findings in the abovementioned studies supported our finding suggesting that using intranasal lidocaine, which blocks the sphenopalatine ganglion, is effective for the treatment of acute migraines.[21, 23, 25]

In contrast, in an RCT, Blenda et al. used 1 mL of 4% lidocaine or normal saline intranasally in split doses 2 minutes apart, and intravenous prochlorperazine for migraine patients. The results showed no evidence of intranasal lidocaine providing rapid relief for migraine headache pain in the ED.[8] In another RCT, Acvu et al. used 1 puff intranasal 10% lidocaine (1 puff = 10 mg) or a saline solution and 10 mg of intravenous metoclopramide for the treatment of migraine patients and showed that intranasal lidocaine was no more efficacious than was a normal saline solution.[11] In our meta-analysis, we found that when migraine patients received an antiemetic treatment, intranasal lidocaine did not provide an add-on effect. The reason for this result is that intravenous prochlorperazine and intravenous metoclopramide are sufficient to treat migraine, which was elucidated by previous RCTs[26–28], and these treatments have been suggested as first-line treatments for migraine patients in certain guidelines.[6, 29] The exact mechanism of antiemetics in relieving migraines is unclear.[30] It has been proposed that blocking dopamine receptors improved some patients’ symptoms in the premonitory phase of a migraine by modifying the transmission of nociceptive signals in cortical and subcortical brain regions and subsequently preventing the occurrence of a headache.[30–32] In contrast, intranasal lidocaine relieved migraines by blocking the sphenopalatine ganglion, which regulates cranial parasympathetic outflow through the release of neuropeptides and subsequently inactivating or desensitizing intracranial nociceptors that contribute to the vasodilation of the cerebral vasculature that produces migraines.[7–9] Additional studies are warranted to elucidate whether intranasal lidocaine and antiemetic medications share similar pathways to control acute migraines.

For clinical applications, oral medications have a delayed onset of activity and may not be appropriate for patients with severe nausea with or without vomiting, and suppositories are inconvenient.[33] Intranasal delivery of headache medications represents an effective alternative to tablets, suppositories, and self-injection. The intranasal route is a beneficial way to deliver headache medication for various reasons. First, intranasal administration bypasses the gastrointestinal tract, absorption from which is slowed during a migraine. Thus, intranasal delivery not only offers a more rapid onset of action than oral medications, but it is also appropriate in patients with nausea and vomiting, which are symptoms that limit or preclude oral administration. Second, intranasal administration prevents the risk for needle-stick injuries and is designed to relieve the potential emotional trauma and pain that may arise from the insertion of an intravenous catheter. Third, since the limited capacity of nasal mucosa to absorb medication reduces the risk of overdosage, intranasal delivery is a safer route of administration than the oral route is. However, the main limitation of this therapy is the difficulty of administration.[34] A lidocaine-soaked swab has to be inserted (or nasal spray has to be administered) via the nostrils with the patient preferably in the supine or sitting position with the neck extended. The swab is then advanced in each nostril after adequate lubrication until resistance is encountered, which is usually provided by the posterior pharyngeal wall superior to the middle turbinate. The swab is left in place for 15–20 min and then removed.[34] The need to position the patient properly while he or she is lying down is the main factor limiting the effectiveness of this therapy.[7] Using 4% lidocaine in a metered-dose spray bottle is a promising alternative method of relieving acute migraines, as it can be carried out by the patient, may be practical, and may be easy to use.[35]

The most common adverse reaction is local irritation.[11] Maizels et al. reported that most side effects are local irritation (burning, stinging, numbness) of the nose or eye; unpleasant taste, gagging, and numbness in the throat; and nausea.[7] No severe adverse effects were reported in the studies included in our study.[7, 8, 11–13, 19] Intranasal lidocaine has an excellent safety profile and is effective in treating acute migraines.[29]

Significant heterogeneity existed among our selected studies, which is attributable to various clinical factors. The time at which analgesics were taken before the patient was admitted to the ED was different across all studies. No studies reported the effect of intranasal lidocaine on subclasses of migraines. The applied volume and concentration of lidocaine and the method of delivering the medication also differed across the studies. Moreover, the differences in the definitions of the outcomes of the included studies may contribute to interstudy heterogeneity.

There are notable limitations to this meta-analysis. First, intranasal lidocaine can cause local irritation, which influences the blinding of participants. Second, many included studies were rated as having some concern of bias because pain is a patient-reported subjective outcome, which may vary among patients in different populations. Third, two studies included were not studies primarily on migraine patients. The statistical power reduced when we extracted data on the subset of migraine patients in these two studies. Fourth, some included studies did not demonstrate the standardized treatment protocols. Fifth, given that only a few studies with relatively small sample sizes were included, overestimation of the results may exist. Finally, publication bias was not assessed due to the limited number of reviews.

In conclusion, the application of intranasal lidocaine can effectively reduce pain intensity, provide many patients with a decrease in the initial pain by 50% or more, and decrease the need for rescue medication without increasing the occurrence of relapse and tolerable adverse events. When patients have received antiemetics as a treatment for migraines, intranasal lidocaine did not provide an add-on effect.

## Supporting information

S1 Checklist PRISMA checklist. (DOC)

S1 Table Search strategy. (DOCX)

S2 Table The exclusion criteria of the included studies. (DOCX)
