# Supplementary material for: Intranasal lidocaine for acute migraine: A meta-analysis of randomized controlled trials
Source: PLoS One. 2019 Oct 23;14(10):e0224285. doi: 10.1371/journal.pone.0224285 (PMC6808552; doi:10.1371/journal.pone.0224285)
Supplement: S1 Table — (DOCX) [file pone.0224285.s002.docx]

**S1Table Search Strategy**

| PubMed (November 09, 2018) | | |
| --- | --- | --- |
| Search | Query | Items found |
| 1 | Search lidocaine | 30884 |
| 2 | Search xylocaine | 31235 |
| 3 | Search headache | 81737 |
| 4 | Search migraine | 35704 |
| 5 | Search transnasal | 3312 |
| 6 | Search intranasal | 25840 |
| 7 | Search (lidocaine) OR xylocaine | 31235 |
| 8 | Search (intranasal) OR transnasal | 28972 |
| 9 | Search (((intranasal) OR transnasal)) AND ((lidocaine) OR xylocaine) | 214 |
| 10 | Search (migraine) OR headache | 98889 |
| 11 | Search (((migraine) OR headache)) AND ((((intranasal) OR transnasal)) AND ((lidocaine) OR xylocaine)) | 55 |
| EMBASE (November 09, 2018) | | |
| 1 | ('migraine'/exp OR migraine OR 'headache'/exp OR headache) AND (intranasal OR transnasal) AND ('lidocaine'/exp OR lidocaine OR 'xylocaine'/exp OR xylocaine) | 319 |
| Cochrane (November 09, 2018) | | |
| 1 | (((migraine) OR headache)) AND ((((intranasal) OR transnasal)) AND ((lidocaine) OR xylocaine)) | 17 |
| Scopus (November 09, 2018) | | |
| 1 | TITLE-ABS-KEY ((((migraine) OR headache)) AND ((((intranasal) OR transnasal)) AND ((lidocaine) OR xylocaine))) | 106 |
