# Supplementary material for: Intranasal lidocaine for acute migraine: A meta-analysis of randomized controlled trials
Source: PLoS One. 2019 Oct 23;14(10):e0224285. doi: 10.1371/journal.pone.0224285 (PMC6808552; doi:10.1371/journal.pone.0224285)
Supplement: S2 Table — (DOCX) [file pone.0224285.s003.docx]

**S2 Table. The exclusion criteria of the included studies**

| Study | Exclusion criteria |
| --- | --- |
| Maizel 1996 [19] | Frequency of severe headache > 1 episode/week; migraine duration > 3 days |
| Maizel 1999 [7] | Headache > 15 days/month; Abortive treatment for migraine > 15 days/month; pregnancy |
| Mohammadkarimi 2014 [13] | Received any medication in the previous 2 hours; penetrating head trauma; any sign of skull base fracture; comorbidity epilepsy |
| Blanda 2001 [8] | Took analgesic medications 2 hours before ED; first ED visit for headache; sign of secondary headache; drug or alcohol abuser; pregnancy; lactation |
| Avcu 2017 [11] | Took analgesic drug within 6 hours before ED; hemodynamic abnormality; sign of meningitis; pregnancy |

Footnote: ED, emergency department;
